# Improving Generalization Based on *l*_1_-Norm Regularization for EEG-Based Motor Imagery Classification

**DOI:** 10.3389/fnins.2018.00272

**Published:** 2018-05-09

**Authors:** Yuwei Zhao, Jiuqi Han, Yushu Chen, Hongji Sun, Jiayun Chen, Ang Ke, Yao Han, Peng Zhang, Yi Zhang, Jin Zhou, Changyong Wang

**Affiliations:** ^1^Department of Neural Engineering and Biological Interdisciplinary Studies, Institute of Military Cognition and Brain Sciences, Academy of Military Medical Sciences Beijing, China; ^2^College of Life Science and Technology, Huazhong Agricultural University Wuhan, China; ^3^Neural Interface & Rehabilitation Technology Research Center, Huazhong University of Science and Technology Wuhan, China; ^4^Stem Cell and Tissue Engineering Lab, Beijing Institute of Transfusion Medicine Beijing, China

**Keywords:** motor imagery, electroencephalography (EEG), classification, *l*_1_-norm regularization, generalization

## Abstract

Multichannel electroencephalography (EEG) is widely used in typical brain-computer interface (BCI) systems. In general, a number of parameters are essential for a EEG classification algorithm due to redundant features involved in EEG signals. However, the generalization of the EEG method is often adversely affected by the model complexity, considerably coherent with its number of undetermined parameters, further leading to heavy overfitting. To decrease the complexity and improve the generalization of EEG method, we present a novel *l*_1_-norm-based approach to combine the decision value obtained from each EEG channel directly. By extracting the information from different channels on independent frequency bands (FB) with l1-norm regularization, the method proposed fits the training data with much less parameters compared to common spatial pattern (CSP) methods in order to reduce overfitting. Moreover, an effective and efficient solution to minimize the optimization object is proposed. The experimental results on dataset IVa of BCI competition III and dataset I of BCI competition IV show that, the proposed method contributes to high classification accuracy and increases generalization performance for the classification of MI EEG. As the training set ratio decreases from 80 to 20%, the average classification accuracy on the two datasets changes from 85.86 and 86.13% to 84.81 and 76.59%, respectively. The classification performance and generalization of the proposed method contribute to the practical application of MI based BCI systems.

## 1. Introduction

Noninvasive brain-computer interface (BCI) based on electroencephalography (EEG) has attracted an increasing interest in recent decades owing to its significant potential in practical applications (Wolpaw et al., 2002; Nicolas-Alonso and Gomez-Gil, 2012). For example, motor imagery EEG (MI-EEG) offers users direct control of different devices such as a wheelchair, quadcopter, or robotic arm (Graimann et al., 2008; Lafleur et al., 2013; Meng et al., 2016) through the modulation of thought without external stimuli. Typical MI-EEG data is composed of multichannel signals recorded from several electrodes placed on the scalp corresponding to the motor-relevant cortex (Blankertz et al., 2008). In order to achieve high classification accuracy, merging of signals from scalp spatial districts is required to suppress the data noise cause by imperfect conductivity of human tissues.

Apparently, as a mirror of the total brain activity in specific regions, multichannel EEG signals interact with each other intrinsically. This interaction is believed to originate from the fundamental mechanism of the information processing within the brain, such as the distributed and co-related function of different cerebral cortex (Baillet et al., 2001). Thus, a specific brain activity is typically mirrored by more than one site on the scalp, leading to considerably redundant information involved in multichannel EEG signals. Moreover, informative EEG features such as task relevant and event-related potentials are likely mixed with blurred features and submerged into the raw data owing to the artifacts and merging effects of the conductive scalp and skull (Pfurtscheller et al., 2006). Due to the insufficient EEG data for classifier training, the complexity of classification algorithms may increase with redundant features involved in EEG signals, adversely affecting their generalization.

In past decades, numerous literatures, such as the common spatial patterns (CSP) (Müllergerking et al., 1999; Ramoser et al., 2000), have focused on this research area. CSP usually amplifies the class disparation in spatial domain by covariance analysis. However, it ignores the task related differences across local regions in frequency domain, which is also important in processing rhythmic activities such as motor imagery EEG. Besides, it may bring in relatively large number of undetermined parameters (the dimensions are the number of output channels multiply by the number of input channels), leading to complicated models, and therefore vulnerable to overfitting especially when the training samples are insufficient.

To avoid this limitation and reduce overfitting during EEG classification, we propose a novel framework named COL (Channel optimization based on *l*_1_-norm). For the sake of mitigating generalization error caused by overfitting, we introduce a sparse l1-norm regularization to solve the optimal weights of channels during combination of each channel's decision value, in which the sparse optimal weights are solved by minimizing the least square error between the predicted labels and the real labels. The optimized model has only a few feature parameters, that is, the channel number, the upper/lower frequency band, and the weight. Benefited from extracting the information from different channels on independent frequency bands with L1-norm regularization, the algorithms proposed fits the training data with much less parameters compared to CSP methods, which enables it to reduce overfitting.

Experimental results on real world datasets demonstrate the effectiveness and robustness of the proposed method, validating its generalization in practical applications.

Our main contributions are highlighted in the following:

- We provide a simple but effective model to reduce overfitting in EEG classification by reducing the number of undetermined parameters.
- We introduce an effective and efficient iterative solution to train the model.
- We demonstrate the superiority of the generalization of our methods on real world datasets.

The remainder of this paper is organized as follows. In section 2, we overview related works. We formulate the proposed method and provide an efficient solution and complexity analysis in section 3. A description of the datasets, the details of the experimental setups, experimental results and discussion are presented in section 4, followed by our conclusions in section 5.

## 2. Related works

Significant efforts have been made in the classification of motor imagery EEG signals. A key point to promote the accuracy of classification algorithms is to prevent overfitting during EEG classification. Here we give a brief review of existing methods for EEG classification from two strategies, and some efforts to reduce overfitting.

Because of the characteristics of different regions of the brain, a number of researches have attempted to process signals from different channels independently. An approach was presented to determine the contribution of different bandwidths of the EEG signal in different recording sites using the multiple kernel learning (MKL) method in Schrouff et al. (2016). Channel-frequency map (CFM) was proposed as a tool to develop data-driven frequency band selection methods for parallel EEG processing in Suk and Lee (2011). Genetic algorithm was utilized to identify individually optimized brain areas and frequency ranges based on a predefined chromosome simultaneously in Lee et al. (2012). Popular deep learning was also introduced in this area. For example, deep belief network (DBN) was employed to reveal the critical frequency bands for emotion recognition (Zheng et al., 2015). Support vector machine (SVM) was considered as a useful method to solve small sample and nonlinear classification problems (Boser et al., 1992). SVM was applied in the feature optimization and classification of MI-EEG (Chatterjee and Bandyopadhyay, 2016; Ma et al., 2016), resulting in a speedup of classification while loss in generalization remained acceptable (Xu et al., 2010). Hybrid spatial finite impulse response (FIR) filters of high-order and data-driven were channel-specifically designed to complement broadband CSP filtering in Yu et al. (2013). In this manner, they facilitate the study of the specific properties of the channels. Nevertheless, their disregard of the interaction among channels likely submerged significant data into irrelevant and redundant signals, negatively influencing the classification performance. Another disadvantage of this approach is the significant computational burden related to the enormous volume of signals.

There have also been several researches that have attempted to address the combination of multichannel EEG data. Well-known CSP methods combined signals from multiple channels by amplifying the class disparity in the spatial domain by covariance analysis (Blankertz et al., 2007; Li et al., 2013). Improved CSPs, such as common spatio-spectral pattern (CSSP) (Lemm et al., 2005), iterative spatio-spectral pattern learning (ISSPL) (Wu et al., 2008), and filter bank common spatial pattern (FBCSP) (Kai et al., 2012) were introduced to optimize the combination of multichannel signals by designing novel spectral weight coefficient evaluation. Another spatial filtering algorithm called discriminative spatial patten (DSP) solved single trial EEG classification by maximizing the between-class separation (Duda et al., 2001; Hoffmann et al., 2006). CSP and DSP were combined to more efficient feature extraction and classification of single trial EEG during finger movement tasks (Liao et al., 2007). In addition to these methods, there are numerous researches focusing on subset selection of EEG channels. Based on grouped automatic relevance determination, group-sparse Bayesian linear discriminant analysis (gsBLDA) was presented to select EEG channels (Yu et al., 2015). The Separability & Correlation (SEPCOR) approach was designed to automatically search for an optimal EEG channel subset with minimum correlation and maximum class separation (Shri and Sriraam, 2016; Student and Sriraam, 2017). Sequential floating forward selection (SFFS) performed a loop of channel selection continuously by iteratively adding and eliminating EEG channels (Pudil et al., 1994; Meng et al., 2011). By considering adjacent channels as one feature according to their distribution on the cerebral cortex, an improved SFFS (ISFFS) was proposed to remove task-irrelevant and redundant channels with low computational burden (Qiu et al., 2016). In order to reduce overfitting, L1 norm regularization was applied in constructing spatial filters for its competence to achieve sparse solution (Silva et al., 2004; Donoho, 2006; Farquhar et al., 2006). Sparse common spatial pattern (SCSP) was applied to optimally select the least number of channels while containing high performance in classification, with l1/l2 norm as the regularization term (Arvaneh et al., 2011). By combining L1 norm based Eigen decomposition into CSP, a L1-norm based CSP was proposed to effectively improve the robustness of BCI system to EEG outliers and achieved higher classification accuracy than the conventional CSP (Li et al., 2013). A modified CSP with l1 sparse weighting method was developed for EEG trial selection, and successfully rejected low-quality trials in a sparsity-aware way (Tomida et al., 2015). These approaches are effective in determining the informative subset or combination weights of channels based on shallow features extracted from voltage signals. However, the CSP in EEG classification generates a spatial filter matrix that generally contains too many parameters, and therefore vulnerable to be overfitting especially when insufficiency training data is available. A model that requires few number of parameters while utilizing the features right related to task will be potential for EEG classification.

## 3. Proposed methods

In this section, we introduce notations used throughout this paper and present the concrete formulation of the proposed method. We then provide a simple yet effective algorithm to solve this problem, followed by an analysis of its computational complexity.

### 3.1. Notations

In this document, scalars, matrices, vectors, sets, and functions are denoted as small, boldface capital, boldface lowercase, fraktur capital, and script capital letters, respectively. **x**^*T*^, **X**^*T*^, **x**_*i*_, **X**_*i*_, **X**_*ij*_, **X**_(*i*,:)_ and **X**_(:,*j*)_ indicate the transpose of vector **x**, the transpose of matrix **X**, the *i*-th element of **x**, the *i*-th sample of the variable **X**, the element of **X** occurring in the *i*-th row and *j*-th column, the *i*-th row of **X** and the *j*-th column of **X** respectively. Moreover, ||**x**||_1_ is the *l*_1_-norm of **x**, ||**X**||_1_ and ||**X**||_2_ are the *m*_1_-norm and *m*_2_-norm of matrix **X**. See the Appendix section for definitions of norm terms.

### 3.2. Problem formulation

In order to reduce the model complexity and the number of undecided parameters, we firstly generate features (rough dichotomous probabilities) of each single channel. Then, features of all channels are selected by sorting each channel according to their Fisher Criterion scores (F-score) (Müller et al., 2004; Duda et al., 2012), denoting distance between the class means in relation to the intra-class variances.

Afterwards, we introduce a *l*_1_-norm regularized sparse least square regression to directly minimize the error between predicted and ground-truth labels, contributing to a simplified model with optimized parameters. The optimized model has much fewer parameters than most existing models.

Assume that we have recorded EEG signals of *N* trials, and let $𝔛={\left\{{\text{X}}_{i}\right\}}_{i=1}^{N}$ be the set of EEG signal corresponding to the *i*-th trial of motor imagery. Specifically, we represent the segment of EEG signals as matrix **X**^*M*×*C*^, where *M* and *C* are the number of sampled time points and channels in a trial respectively. The class indicator vector can be denoted as $\tilde{\text{y}}\in {\left\{0,1\right\}}^{N}$, where ${\tilde{\text{y}}}_{i}=0$ and ${\tilde{\text{y}}}_{i}=1$ indicate that the *i*-th trial is left/right hand and foot motor imagery, respectively.

#### 3.2.1. Extracting and selecting features from each channel

Suppose that all channels are independent from each other, we take a signal vector **x**^*M*×1^ of one channel as an example to define the feature extraction and selection method.

First, we remove the average values channel-wise by applying the common average reference (CAR), which is widely used in EEG preprocessing (Offner, 1950; Wu and Ge, 2013), that is

(1) $$\begin{array}{l} \text{x}\leftarrow \text{x}-\bar{\text{x}}, \end{array}$$

where $\bar{\text{x}}$ is the average over all values of **x** at each channel.

Then, **x** is preprocessed by a band-pass filter. The upper bound *f*_*max*_ and lower bound *f*_*min*_ of the filter is chosen from the frequency list $F=\{f_{0}\theta^{n_{f}}|n_{f}\in \{0,1,...,N_{f}\}\}$, where *f*_0_ is the base frequency, θ is a constant scaling factor, *n*_*f*_ is the selected power coefficient, and *N*_*f*_ is the number of candidate element frequency bands. With the band pass filtered signals, the envelope data is obtained using a discrete-time Hilbert transform, whose complex magnitude, denoted as $\hat{\text{x}}$, is used to replace **x** for further feature extraction.

Afterwards, we extract its feature as

(2) $$\begin{array}{l} \gamma =\log\left(\frac{1}{M}||\hat{\text{x}}|{|}_{2}^{2}\right). \end{array}$$

Next, we determine *f*_*max*_ and *f*_*min*_ by maximizing the F-score (Müller et al., 2004; Duda et al., 2012), which are determined by

(3) $$\begin{array}{l} F\text{-}score=\frac{{\left(\bar{\gamma^{+}}-\bar{\gamma^{-}}\right)}^{2}}{\bar{{\left(\gamma^{+}-\bar{\gamma^{+}}\right)}^{2}}+\bar{{\left(\gamma^{-}-\bar{\gamma^{-}}\right)}^{2}}}, \end{array}$$

where γ^+^ and γ^−^ denote the features of trials labeled “1” or “0,” respectively.

Lastly, the predicted label, the main feature from signal **x** of the current channel, can be obtained by

(4) $$\begin{array}{l} p=S\left(\frac{\delta (\bar{\gamma^{+}}-\bar{\gamma^{-}})}{\sqrt{\Delta_{\gamma }}}\left(\gamma -\frac{\bar{\gamma^{+}}+\bar{\gamma^{-}}}{2}\right)\right), \end{array}$$

where $S\left(x\right)=\frac{1}{1+e^{-x}}$ is the sigmoid function, δ(*x*) is the sign function, and Δ(*x*) is the variance of *x*.

#### 3.2.2. Defining the object of our simplified model

With each element gained from the above section channel-by-channel and trial-by-trial, we could get the feature matrix **P**. Thus, we define the final decision value as **p** = **Pw** + **1**^*T*^*b* ∈ [0, 1]^*N*^, where ${\text{P}}_{ij}^{N\times C}$, **w**^*C*×1^ and **1**^1×*N*^, *b* is the predicted label gained in the *i*-th trial by signals in the *j*-th channel, the weights of *C* channels, a vector of all elements “1” and the bias of all trials. Inspired by the least absolute shrinkage and selection operator (lasso) (Tibshirani, 2011), the object can be written as

(5) $$\begin{array}{l} \min L:\min\frac{1}{2}||\text{p}-\tilde{\text{y}}|{|}_{2}^{2}+\alpha R\left(\text{w}\right), \end{array}$$

or

(6) $$\begin{array}{l} \min L:\min\frac{1}{2}||\text{P}\text{w}+1^{T}b-\tilde{\text{y}}|{|}_{2}^{2}+\alpha R\left(\text{w}\right). \end{array}$$

where α is a balance parameter proportional to *N* and R(w) is a regularization term on **w**.

Clearly, **P** is obtained through the above section. Thereby, the undetermined variables in Equation (6) are **w** and *b*^1^, if we properly define functions R(w).

Recalling that there is frequently redundant and irrelevant channels in practical MI-BCI, we can define R(w) as a sparsity metric on **w**, such as its *l*_1_-norm. Therefore, the optimization problem we must solve can be expressed as

(7) $$\begin{array}{l} \begin{array}{c} \min L\left(\text{w},b\right):\\ \underset{\text{w},b}{\min}\frac{1}{2}||\text{P}\text{w}+1^{T}b-\tilde{\text{y}}|{|}_{2}^{2}+\alpha ||\text{w}|{|}_{1}. \end{array} \end{array}$$

### 3.3. Solution to the formulation

Intuitively, an iterative multiplicative updating procedure is designed to solve Equation (7). In each step, we first fix **w** to determine the optimal *b*, and then solve **w** by fixing *b*.

For Equation (7), we redefine the object of *b* as

(8) $$\begin{array}{l} \begin{array}{c} \min G\left(b\right):\\ \underset{b}{\min}\frac{1}{2}||\text{P}\text{w}+1^{T}b-\tilde{\text{y}}|{|}_{2}^{2}. \end{array} \end{array}$$

It should be noted that the inequality $||\text{A}|{|}_{2}^{2}+||\text{B}|{|}_{2}^{2}\geq \frac{1}{2}||\text{A}+\text{B}|{|}_{2}^{2}$ holds. Thus, we can determine that $||\text{P}\text{w}+1^{T}b_{1}-\tilde{\text{y}}|{|}_{2}^{2}+||\text{P}\text{w}+1^{T}b_{2}-\tilde{\text{y}}|{|}_{2}^{2}\geq 2||\text{P}\text{w}+1^{T}\frac{b_{1}+b_{2}}{2}-\tilde{\text{y}}|{|}_{2}^{2}$, which indicates that G(b) is convex in terms of **b**. Therefore, we have

(9) $$\begin{array}{l} \begin{array}{c} \frac{\partial G\left(b\right)}{\partial b}=1\left(\text{P}\text{w}-\tilde{\text{y}}\right)+11^{T}b\\ =1\left(\text{P}\text{w}-\tilde{\text{y}}\right)+Nb. \end{array} \end{array}$$

Then, by setting $\frac{\partial G\left(b\right)}{\partial b}=0$, we obtain the optimal *b* as

(10) $$\begin{array}{l} b\leftarrow \frac{1\left(\text{P}\text{w}-\tilde{\text{y}}\right)}{N}. \end{array}$$

Similarly, with *b* fixed as in Equation (10), we redefine the object of **w** as

(11) $$\begin{array}{l} \;\min ℋ(w):\\ \;\underset{w}{\min}\frac{1}{2}||Pw+\frac{1^{T}1(Pw-\tilde{y})}{N}-\tilde{y}|{|}_{2}^{2}+\alpha ||w|{|}_{1}\\ \Leftrightarrow \underset{w}{\min}\frac{1}{2}||(P+\frac{1^{T}1P}{N})w-(I_{N}+\frac{1^{T}1}{N})\tilde{y}|{|}_{2}^{2}+\alpha ||w|{|}_{1}. \end{array}$$

where ⇔, **I**_*N*_ denotes equivalence and the *N*-by-*N* identity matrix.

We exploit the gradient descent method to optimize **w** with positive initialization values.

With Equation (11), we have

(12) $$\begin{array}{l} \nabla_{w}=\frac{\partial ℋ(w)}{\partial w}\\ \;={\left(P+\frac{1^{T}1P}{N}\right)}^{T}(P+\frac{1^{T}1P}{N})w-{\left(P+\frac{1^{T}1P}{N}\right)}^{T}(I+\frac{1^{T}1}{N})\tilde{y}\\ \;+\;\alpha \delta (w)\\ \;=(\frac{3{\left(1P\right)}^{T}(1P)}{N}+P^{T}P)w-(P^{T}+\frac{3P^{T}1^{T}1}{N})\tilde{y}+\alpha \delta (w). \end{array}$$

where δ(**w**) is the sign function. Therefore, we have the update rule

(13) $$\begin{array}{l} \text{w}\leftarrow \text{w}-\eta \nabla_{\text{w}}, \end{array}$$

where η > 0 is the step length determined by Algorithm 1, with which the objective value is minimized along the negative gradient direction.

**Algorithm 1:** Algorithm to determine η in Equation (13)

**Input:**
Current **w** and the corresponding value of the object function H(w), as well as the gradient ∇_**w**_.
**Output:**
Step length η.
1: Initialize the linear step length $\eta_{l}=\frac{H\left(\text{w}\right)}{||\nabla_{\text{w}}|{|}_{2}^{2}}$;
2: Compute the maximum step length under the nonnegative constraint **η**_*nn*_ by dividing **w** by ∇_**w**_ element-wise;
3: Preserve all positive elements of **η**_*nn*_ as $\eta_{nn}^{+}$;
4: Set the maximum step length $\eta_{m}\leftarrow \min\left\{\eta_{l},\eta_{nn}^{+}\right\}$;
5: **while** $H\left(\text{w}-\eta_{m}\nabla_{\text{w}}\right)\geq H\left(\text{w}\right)$ **do**
6: Set η_*m*_←η_*m*_/2;
7: **end while**
8: Set $\tilde{\eta }\leftarrow \eta_{m}$ and $\tilde{H}\leftarrow H\left(\text{w}-\eta_{m}\nabla_{\text{w}}\right)$;
9: **if** $H\left(\text{w}-\frac{\eta_{m}}{2}\nabla_{\text{w}}\right)<\tilde{H}$ **then**
10: Update $\tilde{\eta }=\frac{\eta_{m}}{2}$ and $\tilde{H}=H\left(\text{w}-\frac{\eta_{m}}{2}\nabla_{\text{w}}\right)$;
11: **end if**
12: Compute the parabolic approximation parameters using (14) $$\begin{array}{l} \left(\begin{array}{c} a_{\eta }\\ b_{\eta } \end{array}\right)={\left(\begin{array}{cc} \frac{\eta_{m}^{2}}{4} & \frac{\eta_{m}}{2}\\ \eta_{m}^{2} & \eta_{m} \end{array}\right)}^{-1}\left(\begin{array}{c} H\left(\text{w}-\frac{\eta_{m}}{2}\nabla_{\text{w}}\right)-H\left(\text{w}\right)\\ H\left(\text{w}-\eta_{m}\nabla_{\text{w}}\right)-H\left(\text{w}\right) \end{array}\right) \end{array}$$
13: Set $\eta \leftarrow \min\left\{-\frac{b_{\eta }}{2a_{\eta }},\eta_{m}\right\}$;
14: **if** *a*_η_ ≤ 0 **then**
15: Set η = η_*m*_;
16: **end if**
17: **if** $H\left(\text{w}-\eta \nabla_{\text{w}}\right)>\tilde{H}$ **then**
18: Set $\eta =\tilde{\eta }$.
19: **end if**

With positive initialization values, **w** decreases gradually toward zero. Once any element of **w** reaches zero, signals from the corresponding channel are removed, and the updating in terms of this channel is terminated.

Thus, with a new trial, we can first preprocess the EEG data using band-pass filtering and average removing according to the channel-specific *f*_*min*_ and *f*_*max*_. Then, features are extracted using Equation (2), followed by obtaining the decision value channel-wise using Equation (4). Then, the combined predicted label *p* is computed with learnt **w** and *b*.

It should be noted that the combined predicted label can exceed [0, 1], hence, we define another sigmoid function to normalize this as

(15) $$\begin{array}{l} p_{normal}=S\left(\beta (p-0.5)\right), \end{array}$$

where β is a constant and we fix β = 4 to set the derivative on *p* = 0.5 as “1”.

### 3.4. Flowchart of algorithm

Based on the above analysis, we summarize the detailed optimization algorithm of COL in Algorithm 2.

**Algorithm 2:** Algorithm to Solve COL

**Input:**
*N* EEG data matrices $X_{1}^{M\times C}$, $X_{2}^{M\times C}$,…, and $X_{N}^{M\times C}$; balance parameter α; number of candidate element frequency bands *N*_*f*_; base frequency *f*_0_ and constant scaling factor θ.
**Output:**
Weight vector of *C* channels **w**; bias of *N* trials *b*.
1: **for** *c* = 1, 2, …, *C* **do**
2: Remove average values from EEG signals channel-wise using Eq.(1);
3: Initialize *n*_*f*_*min*__ = 0 and *n*_*f*_*max*__ = 1;
4: Set $f_{min}=f_{0}\theta^{n_{f_{min}}}$ and $f_{max}=f_{0}\theta^{n_{f_{max}}}$;
5: Filter signals by band pass defined by *f*_*min*_ and *f*_*max*_;
6: Obtain the envelope data using a discrete-time Hilbert transform;
7: Extract features using Eq.(2);
8: Compute F-Score using Eq.(3), save it as the temporal best *FS*_*best*_, store *f*_*min*_ and *f*_*max*_;
9: **for** *n*_*f*_*min*__ = 1, 2, 3, …, *N*_*f*_ − 1 **do**
10: **for** *n*_*f*_*max*__ = *n*_*f*_*min*__, *n*_*f*_*min*__ + 1, …, *N*_*f*_ **do**
11: Set $f_{min}=f_{0}\theta^{n_{f_{min}}}$ and $f_{max}=f_{0}\theta^{n_{f_{max}}}$;
12: Filter signals by band pass defined by *f*_*min*_ and *f*_*max*_;
13: Extract features using Eq.(2);
14: Compute F-Score using Eq.(3) as *FS*;
15: **if** *FS* > *FS*_*best*_ **then**
16: Store *f*_*min*_ and *f*_*max*_;
17: Update *FS*_*best*_←*FS*;
18: **end if**
19: **end for**
20: **end for**
21: Filter signals by band pass defined by *f*_*min*_ and *f*_*max*_;
22: **end for**
23: Obtain the decision matrix **P** element-wise using Eq.(4);
24: Initialize **w** and *b*.
25: **repeat**
26: Fix **w**, and update *b* using Eq.(10);
27: Fix *b*, and update **w** using Eq.(13);
28: **until** Convergence criterion satisfied.

### 3.5. Complexity analysis

In this subsection, we analyze the time complexity of Algorithm 2. For all channels, searching for *f*_*min*_ and *f*_*max*_ requires $O\left(MNCN_{f}^{2}\right)$. Computing **P** requires *O*(*MNC*) time. Further, in each iteration for optimizing **w** and *b*, updating **w** requires *O*(*NC*^2^) and *b* requires *O*(*NC*) time. Thus, it requires *O*(*TNC*^2^) to update **w** and *b*. Therefore, the overall cost for Algorithm 2 is $O\left(MNCN_{f}^{2}+TNC^{2}\right)$, where *T* is the number of iterations.

## 4. Experiments

We compared the proposed COL with several classical state-of-the-art methods in terms of the classification and generalization performance. The experimental setups and results are presented in this section.

### 4.1. Experimental setups

#### 4.1.1. Datasets

In our experiments, we selected two public real world datasets. A brief description of these datasets is provided below and their statistics are summarized in Table 1.

**Table 1:** Statistics of the 2 datasets.

**Datasets**	**No. of channels**	**Sampled frequency (Hz)**	**No. of Subjects**	**No. of trials per class**
DS1	118	100	5	140
DS2	59	100	4	100

**DS1**: Dataset IVa from BCI Competition III is a public dataset provided by the Berlin BCI group Fraunhofer FIRST (Intelligent Data Analysis Group) and Campus Benjamin Franklin of the Charité University (Neurophysics Group). This public dataset is recorded from five healthy subjects during right hand and right foot motor imageries. The EEG recordings consist of 118 channels at positions of the extended international 10/20-system. We chose a version of the data that was downsampled at 100 Hz for analysis. In the experiments, subjects performed three motor imageries for 3.5 s after visual cues for left hand, right hand, or right foot. After the duration of motor imagery, a resting interval with random length of 1.75–2.25 s was inserted for relaxation. The dataset provided only EEG trials for right hand and right foot imagery. For each subject, the dataset consisted of signals of 140 trials per class.

**DS2**: Dataset I from BCI Competition IV is a public dataset provided by the Berlin BCI group Fraunhofer FIRST (Intelligent Data Analysis Group) and Campus Benjamin Franklin of the Charité University (Neurophysics Group). This public dataset is recorded from four healthy subjects during two classes of motor imagery selected from three classes: left hand, right hand, and foot (side chosen by the subject; optionally also both feet). In the experiment, the data was continuous signals of 59 EEG channels and visual cues pointing left, right or down were presented for a period of 4.0 s during which the subject was instructed to perform the cued motor imagery task. These periods were interleaved with 2.0 s of blank screen and 2.0 s with a fixation cross displayed in the center of the screen. The dataset provided only EEG trials for left hand and foot imagery. For each subject, the dataset consisted of signals of 100 trials per class.

Since the band-pass filtering is involved in the proposed method, the pre-processing in the experiment is mainly two data normalization. The first one is removing the direct component in each trial, and the second one is normalizing all channels with mean zero by subtracting the mean values of each channel.

#### 4.1.2. Baselines

To validate the effectiveness of the proposed COL, we compared it with the following feature selection and optimization methods.

1. **All channels**: Signals of all available channels are used for EEG classification;
2. **3C channels**: Signals of C3, Cz, and C4 are used for EEG classification;
3. **gsBLDA** (Yu et al., 2015): Signals of channels selected based on group Bayesian linear discriminant analysis are used for EEG classification;
4. **MRCS** (Zhang et al., 2016): Signals of channels selected by combining ReliefF and SVM are used for EEG classification;
5. **CSTI** (Yang et al., 2016): A subject-specific channel selection method based on a criterion, called F score, to realize the parameterization of both time segment and channel positions;
6. **NSGA-II** (Kee et al., 2015): Signals of channels selected by a multi-objective genetic algorithm, i.e., NSGA-II, are used for EEG classification.

In order to ensure that the performance comparison mainly focused on the feature selection and optimization abilities of different algorithms, we used the same training and testing partitions for all methods when performing the cross-validation. We used autoregression analysis (AR) (Pfurtscheller et al., 1998) for EEG feature extraction after channel optimization by these comparison algorithms, except for the CSTI which contained the feature extraction procedure in its framework. Then linear discrimination analysis (LDA) was used for classification.

#### 4.1.3. Parameter settings

It is universally acknowledged that the performance of the majority of methods depends on their parameters. Therefore, we set the parameters used in our experiments in advance. As EEG is recorded continuously, it is necessary to choose a time interval to cut signals into a specific duration. In this work, we selected different durations of data from 3.5 s of continuous motor imagery for data processing and classification for DS1 and 4 s for DS2, namely 0–2, 0–2.5, 0.5–2.5, 0.5–3, 1–3, 1–3.5, 1.5–3.5, 1.5–4, and 2–4 s. Except for the results on different time intervals, we used signals in the time interval of 0.5-3 s for each trial on DS1, and that of 0–4 s for each trial on DS2. Moreover, α, *N*_*f*_, *f*_0_, and θ were set to 0.01, 9, 7, and 1.22, respectively. Further, we defined two convergence criteria in Algorithm 2; the iteration terminated if either of them was satisfied. The first one is $H\left(\text{w}-\eta \nabla_{\text{w}}\right)>0.9999H\left(\text{w}\right)$, indicating the updating of **w** is almost stopped. The second was that the number of iteration met the maximum iterations, which was set to 1,000 in this work. All trials of the DS1 were used to perform cross validation. Only the calibration data of DS2 were used, since these data were provided with complete marker information. Except for the experiments on different sizes of training sets, eighty percent of the data were used for training data, the remaining part were used for testing the data in each fold. Then, five-fold cross validation was repeated five times and the accuracies were averaged to obtain the mean result of the five-fold cross validation.

### 4.2. Results

**Figure 1:**
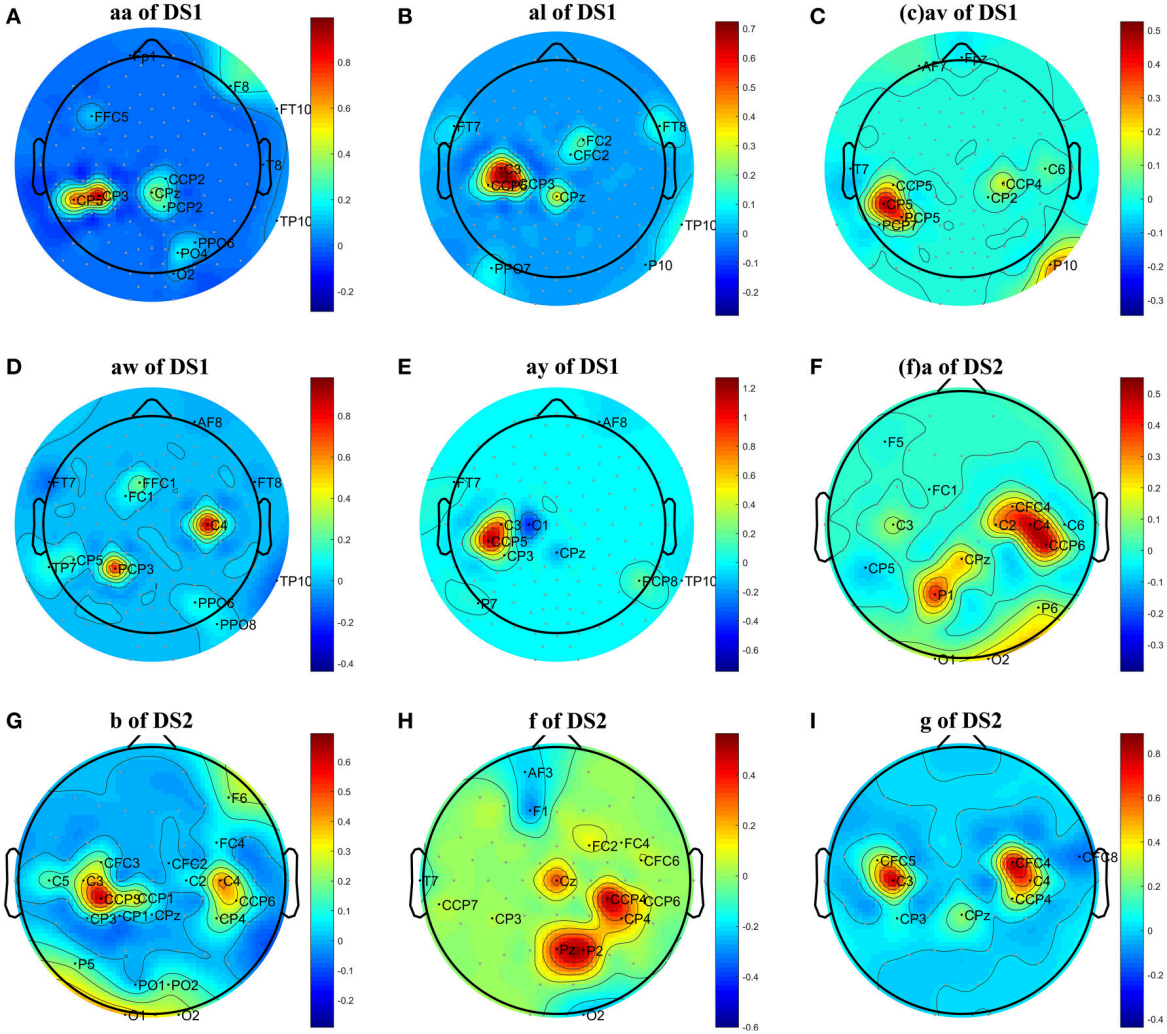
Topographical map of optimal channels and weights on subjects: **(A)** aw of DS1, **(B)** av of DS1, **(C)** al of DS1, **(D)** aa of DS1, **(E)** ay of DS1, **(F)** a of DS2, **(G)** b of DS2, **(H)** f of DS2, **(I)** g of DS2.

**Figure 2:**
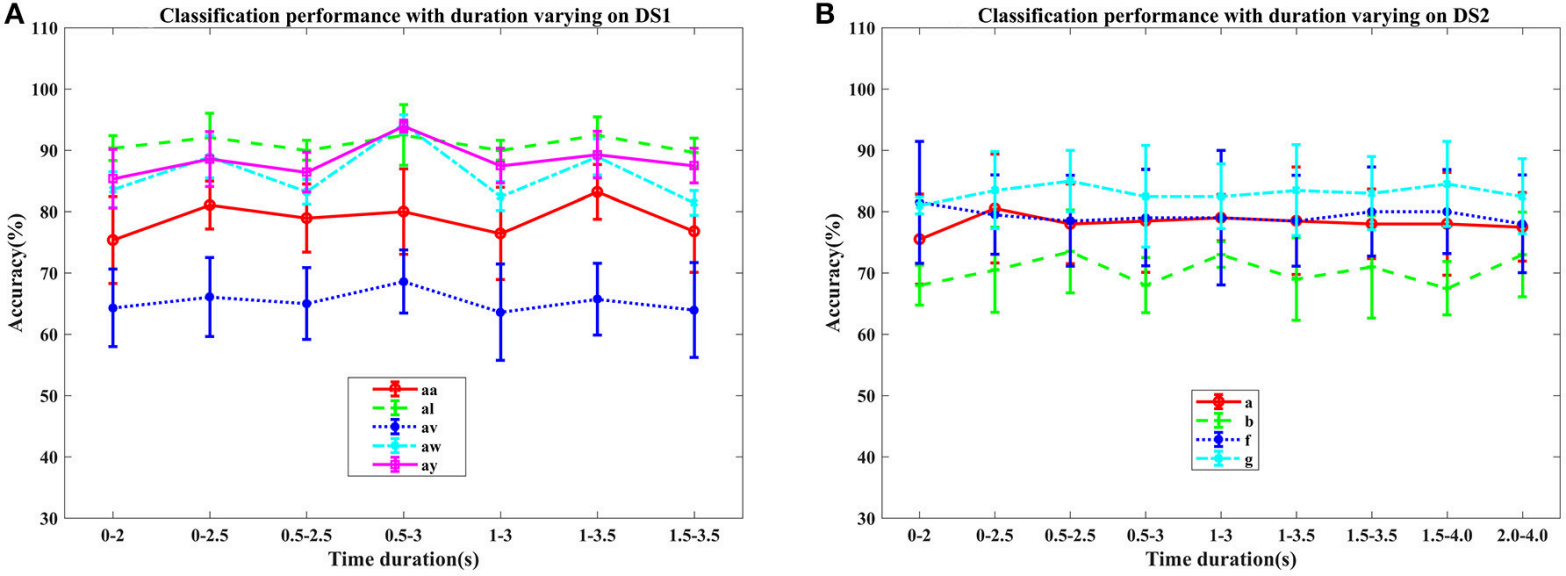
Average classification accuracy (%) and standard deviation of COL with signal time duration varying on nine subjects: **(A)** aa, al, av, aw, and ay of DS1, and **(B)** a, b, f, and g of DS2. Each line type represents a subject's mean and std. of classification accuracy at different time durations.

**Figure 3:**
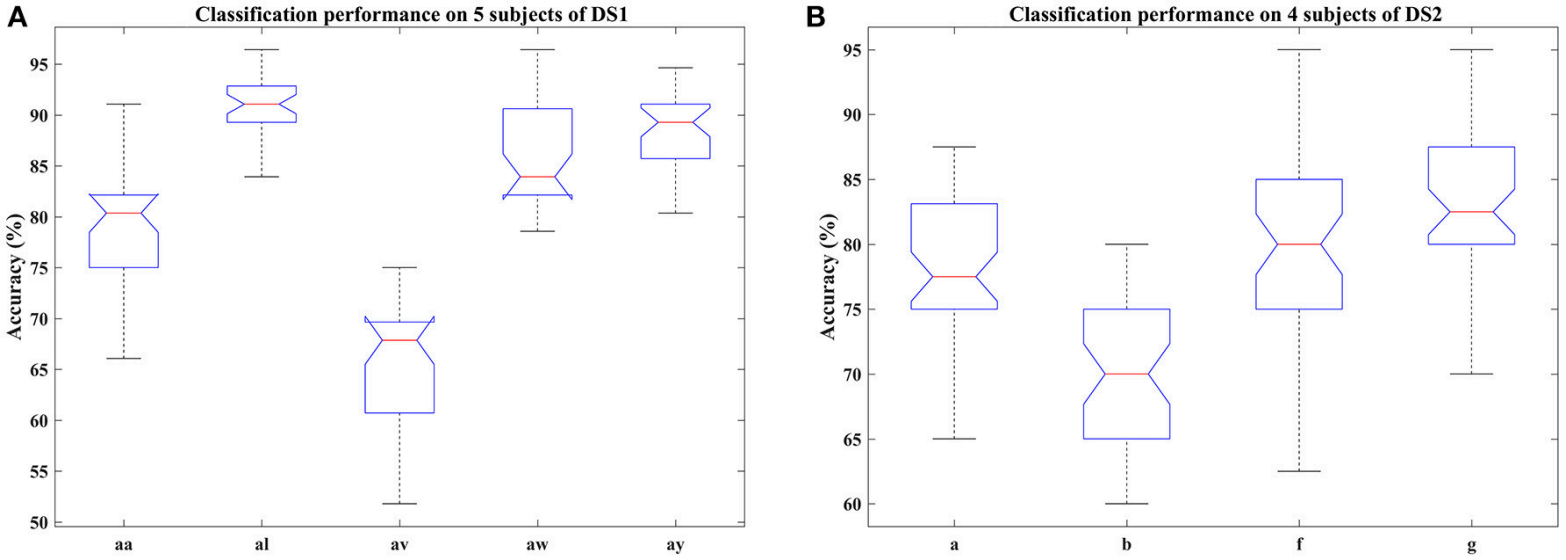
Classification performance of COL on nine subjects: **(A)** aa, al, av, aw, and ay of DS1, **(B)** a, b, f, and g of DS2. Blue box indicates the range from 25 and 75% of the accuracy distribution, red line marks the median of the accuracy, notches in the box indicate the variability of the median between samples at the 5% significance level, extended whisker is the furthest observations in the range of 1.5 times the interquartile range away from the top or bottom of the box.

To examine the effectiveness of the proposed COL, the classification on the two-class MI-EEG experimental results are given and analyzed in this section. We first present the optimal frequency bands with nonzero weights of the channels subject-specifically in Table 2 and Figure 1. The performance of the proposed COL on different signal time duration and subjects is displayed in Figures 2, 3. We discuss the sensitivity of the proposed COL on different sizes of training sets in **Figure 5**. Further comparison and analysis of these results with several channel selection algorithms in literature (Kee et al., 2015; Yu et al., 2015; Yang et al., 2016; Zhang et al., 2016) are provided in Tables 3, **5**, **9**, **10**.

**Table 2:** OFB of channels with non-zero weights on subject ay of DS1.

**Channel number**	**Channel location**	***f*_*min*_**	***f*_*max*_**
60	CCP5	18.92	23.08
52	C3	8.54	34.35
70	CP3	10.42	28.16
87	P7	15.51	34.35
32	FT7	10.42	12.71
53	C1	10.42	34.35
9	AF8	8.54	12.71
85	PCP8	28.16	34.35
72	CPz	23.08	34.35
77	TP10	10.42	12.71

**Table 3:** The mean classification accuracy and standard deviation (%) of 5 cross-validation with the number of corresponding channels (listed in the bracket) of COL and baselines on 5 subjects of DS1.

**Method**	**Subject**					**Mean**	**Significant**
	**aa**	**al**	**av**	**aw**	**ay**		
BS1	71.43	83.57	65.71	76.07	78.57	75.07	^****^
	± 4.37	± 5.56	± 8.41	± 4.11	± 3.34		
BS2	69.29	91.07	58.93	79.29	85.36	76.79	^****^
	± 5.84	± 2.19	± 3.79	± 2.99	± 6.61		
gsBLDA	70.36(76)	87.86(70)	68.21(101)	80.00(118)	77.50(97)	76.79	^****^
	± 4.11	± 3.19	± 6.61	± 4.62	± 6.87		
MRCS	75.00(94)	84.64(89)	66.07(115)	77.50(94)	79.64(86)	76.57	^****^
	± 1.26	± 5.45	± 7.14	± 2.71	± 7.21		
CSTI	74.64(8)	**94.64(20)**	**72.50(18)**	81.79(16)	92.86(12)	83.29	ns
	± 4.62	± 1.26	± 8.80	± 4.07	± 6.92		
NSGA-II	75.36(55)	85.36(55)	65.00(51)	76.43(71)	77.50(64)	75.93	^****^
	± 6.11	± 4.96	± 3.70	± 4.62	± 8.43		
L2-norm	73.57(72.4)	87.86(74)	61.79(77)	83.57(76.6)	81.79(75.2)	77.71	^****^
	± 2.93	± 3.43	± 4.66	± 4.45	± 4.62		
Proposed	**80.00(13.4)**	92.50(12.2)	68.57(12)	**94.29(11.2)**	**93.93(9.8)**	**85.86**	–
	± 6.96	± 4.96	± 5.14	± 1.49	± 0.98		

*The best performance is highlighted in bold. Performance of BS1 and BS2 indicates classification accuracy with all channels and 3C channels, respectively. Performance of L2-norm indicates classification accuracy with L2 norm replacing L1 norm as the regularization term*.

**** *p < 0.0001, ordinary one-way ANOVA with Dunnett's multiple comparisons test*.

#### 4.2.1. Results on OFB and weights of channels

We first analyzed the OFB and weights obtained by the proposed COL in this subsection. The results were presented in Table 2 and Figure 1. Table 2 listed the selected channels with different weights on subject ay, as well as their locations on the scalp and optimal *f*_*min*_, *f*_*max*_. In Figure 1, the weights of all channels were reported in the topographical map, where pseudo-red regions were selected channels with large positive weights. Among the total number of 118 channels in DS1 and 59 channels in DS2, the COL selected a dozen of optimal channels for feature classification, leading to a simplified model.

Table 2 demonstrated the capability of the proposed COL to determine the OFB for each channel independently. It was beneficial for exploring and exploiting sensitive frequencies of different cerebral regions during motor imagery tasks. Moreover, an interesting observation from this table was that these OFBs were all approximately among μ and β rhythm, which was believed to be closely related to motor imagery.

From Figure 1, we have three main observations. First, the significant channels selected by COL exhibited a physiologically interpretable topography, where the regions near the mid-central vertex and left hemisphere were pivotal to discriminating the foot and right-hand imagery. Secondly, the weights obtained by COL were heavily concentrated on one or two regions, and signals from the majority of the channels were discarded, which contributed to reduce the computational costs and overfitting. Thirdly, significant regions for subject av of DS1 and subject b of DS2 were marginally farther from the vertex than other subjects, not totally focusing on the sensorimotor cortex, which could adversely influence the MI classification accuracy.

#### 4.2.2. Performance on different signal time duration and subjects

In this section, we examined the classification performance of COL on different signal time durations and subjects through five-fold cross-validation. The accuracies with respect to the selection of time interval and subjects were displayed in Figures 2, 3. Figure 2 plotted the mean accuracies and standard deviation with different signal time durations. In Figure 3, the distribution of the classification accuracies on the five subjects were displayed in box figures, where the tops and bottoms of the blue boxes were the 25th and 75th percentiles of the samples of the accuracy distribution, the red line marked the median of the accuracy, notches in the boxes indicated the variability of the median between samples at the 5% significance level, the extended whisker was the furthest observations in the range of 1.5 times the interquartile range away from the top or bottom of the box.

From these two figures, we can make the following observations. For DS1, higher accuracies were frequently achieved by 2.5 s intervals, instead of 2 s intervals, indicating that more data was beneficial for improving the performance. Specifically, the interval of 0.5–3 s provided the best classification accuracies, approximately. For DS2, the proposed COL got a more stable classification accuracy across different time durations. Accuracy distributions differed among all nine subjects and the proposed COL could determine a relatively stable classification accuracy for the majority of the subjects except for av of DS1 and b of DS2.

We also compared the proposed COL with several classical and state-of-the-art methods; the results were presented in Tables 3, **5**. Each table summarized the mean accuracies of the different methods. The standard deviation and the number of selected channels were also reported. And the F1 score of these methods were also presented for further evaluating the classification accuracy in Tables 4, **6**. The best values were highlighted in bold.

**Table 4:** The mean F1 score and standard deviation (%) of 5 cross-validation of COL and baselines on 5 subjects of DS1.

**Method**	**Subject**					**Mean**
	**aa**	**al**	**av**	**aw**	**ay**	
BS1	70.33	82.81	65.70	76.24	78.12	74.64
	± 4.77	± 5.81	± 8.35	± 4.30	± 3.12	
BS2	68.32	90.71	58.42	77.36	84.30	75.82
	± 5.69	± 2.25	± 3.17	± 3.91	± 7.87	
gsBLDA	67.24	87.07	66.34	79.23	75.44	75.06
	± 7.30	± 3.90	± 8.72	± 5.04	± 8.22	
MRCS	74.40	83.74	66.77	77.59	78.48	76.20
	± 1.59	± 6.12	± 6.33	± 2.19	± 8.19	
CSTI	73.47	**94.42**	**73.47**	81.91	93.09	83.27
	± 4.35	± 1.31	± 9.43	± 3.45	± 6.55	
NSGA-II	73.92	83.99	63.52	77.01	75.73	74.83
	± 6.37	± 5.84	± 5.47	± 3.70	± 10.28	
L2-norm	71.86	87.45	59.63	83.19	81.29	76.69
	± 3.89	± 4.14	± 4.66	± 4.17	± 4.84	
Proposed	**79.38**	92.52	68.33	**94.27**	**93.81**	**85.66**
	± 7.63	± 4.81	± 5.15	± 1.57	± 1.01	

*The best performance is highlighted in bold.Performance of BS1 and BS2 indicates classification accuracy with all channels and 3C channels, respectively. Performance of L2-norm indicates classification accuracy with L2 norm replacing L1 norm as the regularization term*.

Tables 3, 4 indicated that COL achieved the best performance compared with two baselines and four state-of-the-art methods. For the two excellent subjects aw and ay, the proposed COL maintained a relatively stable classification accuracy, indicated by the small standard deviations. Notably, the number of selected channels of COL outperformed the other methods in the vast majority of cases. One-way ANOVA with Dunnett's multiple comparisons test showed that the improvement of COL was significant (*p* < 0.0001) except for CSTI. With regard to the other methods, the proposed COL not only achieved superior accuracy but also preserved fewer channels, demonstrating the superior performance of the proposed method over the state-of-the-art methods. The proposed COL was not the best discriminating right hand and foot imagery on subject al, and av, but the best at classifying the signals of aa, aw, and ay.

Tables 5, 6 illustrated that COL was more than 10% superior to the comparison algorithms on performance. Specifically, COL could discriminate motor imagery of subject b and g with an improvement of 11 and 15% in accuracy over the best baselines, respectively. One-way ANOVA with Dunnett's multiple comparisons test showed that the improvement of COL was significant (*p* < 0.0001). The results apparently verified the positive effect of the proposed channel-optimization strategy on MI-EEG classification. The average number of selected channels of COL maintained the same level in DS1, contributing to a simplified model with optimized parameters.

**Table 5:** The mean classification accuracy and standard deviation (%) of 5 cross-validation with the number of corresponding channels (listed in the bracket) of COL and baselines on 4 subjects of DS2.

**Method**	**Subject**				**Mean**	**Significant**
	**a**	**b**	**f**	**g**		
BS1	74.50	67.00	62.00	72.00	68.88	^****^
	± 3.26	± 4.81	± 4.81	± 5.97		
BS2	69.00	61.00	65.00	77.50	68.13	^****^
	± 6.02	± 3.79	± 5.59	± 3.95		
gsBLDA	74.00 (59)	68.50 (46)	68.00 (3)	74.00 (7)	71.13	^****^
	± 6.52	± 1.37	± 8.91	± 8.02		
MRCS	74.50 (59)	67.00 (59)	63.00 (58)	76.00 (36)	70.38	^****^
	± 3.26	± 4.81	± 4.11	± 2.85		
CSTI	82.50 (21)	69.00 (9)	76.00 (16)	73.50 (19)	75.25	^****^
	± 8.10	± 7.20	± 5.18	± 4.54		
NSGA-II	73.50 (38)	65.00 (34)	67.50 (36)	70.50 (8)	69.13	^****^
	± 5.18	± 10.75	± 1.77	± 8.55		
L2-norm	84.00 (35.2)	66.50 (33.2)	71.50 (30.8)	74.00 (28.6)	74.00	^****^
	± 8.77	± 7.42	± 4.54	± 14.85		
Proposed	**87.50** **(13.2)**	**80.00** **(19.8)**	**84.50** **(12)**	**92.50** **(10.4)**	**86.13**	–
	± 9.19	± 7.50	± 5.70	± 3.54		

*The best performance is highlighted in bold. Performance of BS1 and BS2 indicates classification accuracy with all channels and 3C channels, respectively. Performance of L2-norm indicates classification accuracy with L2 norm replacing L1 norm as the regularization term*.

**** *p < 0.0001, ordinary one-way ANOVA with Dunnett's multiple comparisons test*.

**Table 6:** The mean F1 score and standard deviation (%) of 5 cross-validation of COL and baselines on 4 subjects of DS2.

**Method**	**Subject**				**Mean**
	**a**	**b**	**f**	**g**	
BS1	74.09	67.95	60.77	71.51	68.58
	± 2.60	± 4.34	± 8.73	± 7.26	
BS2	66.70	62.08	62.65	76.57	67.00
	± 5.44	± 3.37	± 8.19	± 4.63	
gsBLDA	73.52	68.63	68.41	73.58	71.04
	± 6.30	± 1.34	± 9.49	± 8.33	
MRCS	74.09	67.95	61.95	75.56	69.89
	± 2.60	± 4.34	± 7.91	± 4.12	
CSTI	82.05	68.46	75.57	74.47	75.14
	± 8.82	± 6.98	± 6.25	± 3.65	
NSGA-II	72.66	64.50	66.02	67.97	67.79
	± 5.66	± 10.76	± 5.66	± 12.10	
L2-norm	83.87	62.60	71.36	73.28	72.78
	± 8.74	± 11.46	± 3.73	± 14.81	
Proposed	**87.00**	**79.20**	**84.49**	**92.38**	**85.77**
	± 10.12	± 7.71	± 5.66	± 3.61	

*The best performance is highlighted in bold. Performance of BS1 and BS2 indicates classification accuracy with all channels and 3C channels, respectively. Performance of L2-norm indicates classification accuracy with L2 norm replacing L1 norm as the regularization term*.

**Figure 4:**
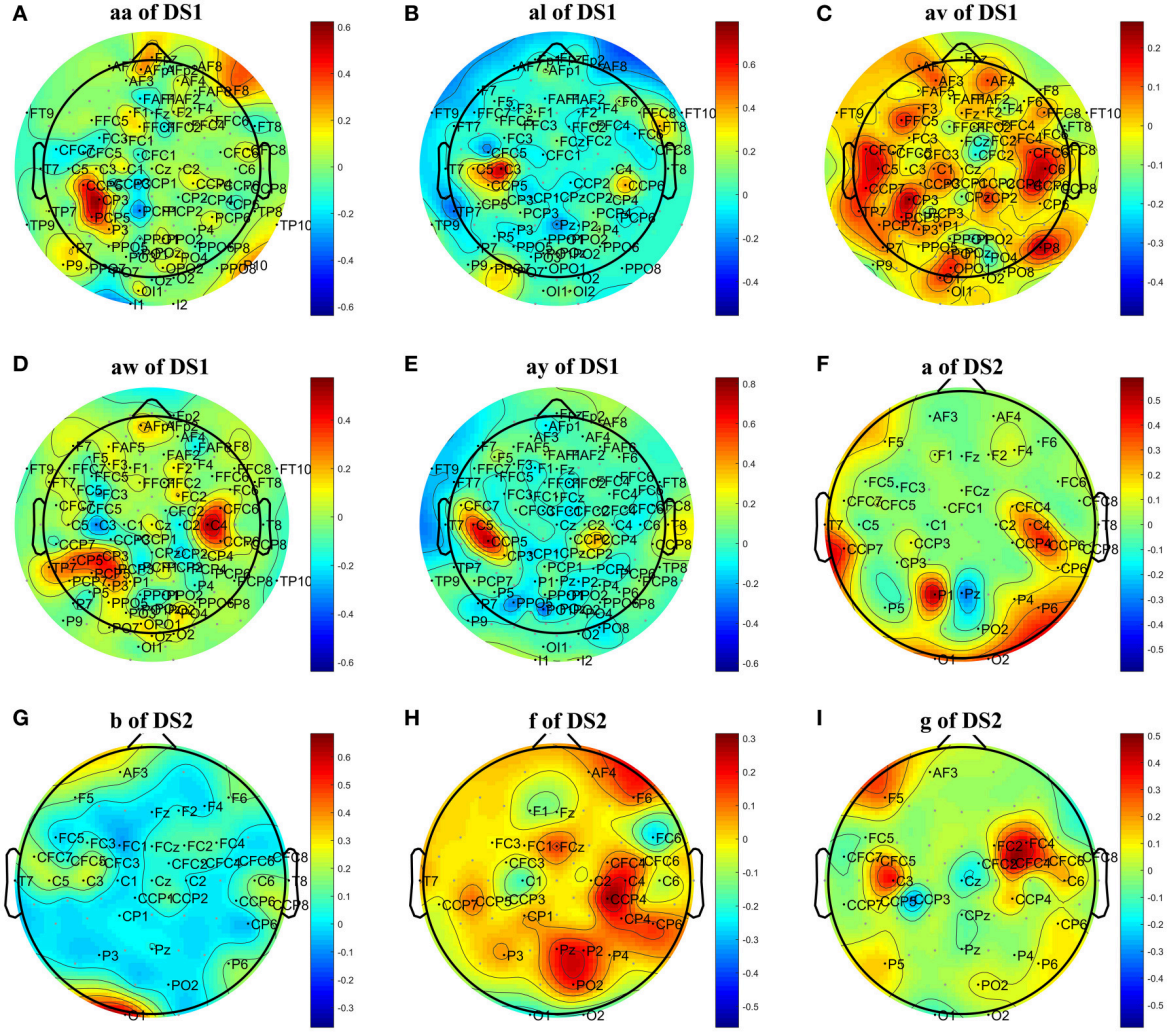
Topographical map of optimal channels and weights with L2 norm regularization term on subjects: **(A)** aw of DS1, **(B)** av of DS1, **(C)** al of DS1, **(D)** aa of DS1, **(E)** ay of DS1, **(F)** a of DS2, **(G)** b of DS2, **(H)** f of DS2, **(I)** g of DS2.

To further investigate the effect of L1 norm on the simplification and classification accuracy of COL, we replaced the L1 norm by a similar L2 norm regularization term and compared its influence on COL's performance. The L1 norm and L2 norm are two different sparse strategies. In comparison, the L1 norm can achieve sparser optimization, and the optimization process is more robust and less susceptible to interference from signal changes, noise, and other factors (Li et al., 2013; Peterson et al., 2015). Tables 3, 5 showed that the optimization based on L2 norm did not result in a simplified classification model. The number of optimal channels was 3–6 times higher than that based on L1 norm. The topographical maps of optimal channels and weights with L2 norm regularization term were also plotted in Figure 4, showing that many redundant channels were selected by the L2 norm regularization. As a result, the classification accuracy obtained by L2 norm was lower than the L1 norm. These results proved that L1 norm played an important role in the optimization of the COL model.

Considering EEG reference may have a fundamental impact on the result, we performed EEG zero-reference by means of the reference electrode standardization technique (REST, Yao, 2001, 2017), which can be found at www.neuro.uestc.edu.cn/rest (Dong et al., 2017). Comparison of the classification results before and after REST processing were provided in Tables 7, 8. We found that the classification performance didn't change significantly after REST processing, except for subject ay of DS1 and subject g of DS2, who received a decrease of approximate to 10% in classification accuracy. The number of optimal channels maintained the same level as before. We infer that the L1 norm-based sparse channel optimization and combination procedure may be insensitive to the detailed characteristics of the signal. As a result, the EEG signal obtained by the simple CAR preprocessing or the more accurate zero-reference preprocessing does not cause apparent difference in the classification performance.

**Table 7:** The mean classification accuracy and standard deviation (%) of 5 cross-validation with the number of corresponding channels (listed in the bracket) of COL and baselines on 5 subjects of DS1 after using REST for zero-reference.

**Method**	**Subject**					**Mean**
	**aa**	**al**	**av**	**aw**	**ay**	
BS1	77.50	85.00	60.71	81.43	78.21	76.57
	± 3.48	± 5.14	± 4.19	± 4.30	± 3.43	
BS2	64.64	88.93	66.79	77.50	82.14	76.00
	± 9.14	± 5.11	± 4.30	± 6.63	± 4.37	
gsBLDA	77.50(118)	87.50(97)	63.57(85)	83.21(95)	80.00(105)	78.36
	± 3.48	± 4.55	± 4.82	± 3.70	± 2.33	
MRCS	77.50(118)	85.71(112)	63.93(108)	82.50(100)	78.21(118)	77.57
	± 3.48	± 3.99	± 5.84	± 6.24	± 3.43	
CSTI	73.21(8)	88.57(31)	**68.93(12)**	75.00(19)	59.64(93)	73.07
	± 4.55	± 4.48	± 7.64	± 3.79	± 7.32	
NSGA-II	76.43(55)	84.29(59)	64.29(35)	85.00(68)	78.93(64)	77.79
	± 4.62	± 4.07	± 6.31	± 4.30	± 5.70	
Proposed	**77.86(15.8)**	**91.43(14.6)**	68.57(11.4)	**86.43(15)**	**83.57(7.6)**	**81.57**
	±5.30	±2.65	±5.30	±2.71	±4.26	

*The best performance is highlighted in bold. Performance of BS1 and BS2 indicates classification accuracy with all channels and 3C channels, respectively*.

**Table 8:** The mean classification accuracy and standard deviation (%) of 5 cross-validation with the number of corresponding channels (listed in the bracket) of COL and baselines on 4 subjects of DS2 after using REST for zero-reference.

**Method**	**Subject**				**Mean**
	**a**	**b**	**f**	**g**	
BS1	70.50	67.00	68.50	63.50	67.38
	± 8.55	± 8.18	± 5.48	± 6.75	
BS2	64.00	54.50	62.00	75.00	63.88
	± 3.35	± 6.71	± 10.22	± 8.84	
gsBLDA	71.50(55)	68.00(58)	68.50(59)	66.00(57)	68.50
	± 10.40	± 7.79	± 5.48	± 8.59	
MRCS	72.00(55)	67.50(58)	70.50(43)	72.00(3)	70.50
	± 6.47	± 7.29	± 4.11	± 3.71	
CSTI	78.00(12)	66.50(32)	75.00(14)	75.00(10)	73.63
	± 5.70	± 7.42	± 7.07	± 4.68	
NSGA-II	72.50(27)	68.00(33)	68.00(27)	73.00(9)	70.38
	± 10.0	± 4.47	± 6.22	± 6.47	
Proposed	**86.00(13.4)**	**77.00(17.8)**	**87.00(12.0)**	**82.50(11.0)**	**83.13**
	±3.79	±6.94	±6.47	±7.91	

*The best performance is highlighted in bold. Performance of BS1 and BS2 indicates classification accuracy with all channels and 3C channels, respectively*.

#### 4.2.3. Results on different sizes of training sets

**Figure 5:**
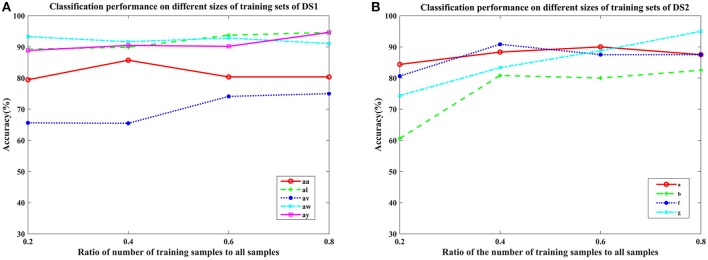
Average classification accuracy of COL with the ratio of the number of training samples to all samples varying from 0.2 to 0.8 with step 0.2 on nine subjects: **(A)** aa, al, av, aw, and ay of DS1, and **(B)** a, b, f, and g of DS2. Each line type represents a subject's classification accuracy at different training samples.

To further verify the generalization of the proposed COL, we plotted the mean classification accuracy with the ratio of the number of training samples to all samples varying from 0.2 to 0.8 with step 0.2 in Figure 5. It appears that the performance of the proposed COL was relatively stable over a large range of the ratio on DS1. Even using only 20% of the samples for training, the accuracy was >90% for subject aw. Furthermore, this result implied that the proposed approach could leverage the supervision information of small numbers of training sets to predict labels of a considerably larger quantity of testing samples. This favorable generalization based on the small sample training complemented the rapid implementation and application practically of the proposed COL. For subject b and g of DS2, an obvious improvement of approximately 20% with training samples increasing from 20 to 80 could be observed. It clearly indicated that COL was capable of leveraging supervised labels to acquire more appropriate undetermined parameters.

The generalization of COL was compared with other mentioned methods above by evaluating the classification performance under small training sets (20% samples for training), as shown in Tables 9, 10. Compared with Table 3, results in Table 9 illustrate that COL was relatively stable with small training sets on DS1, while the other methods indicated a decrease approximate to 9% when training sets became small. The generalization of COL on DS2 did not outperform the others, possibly due to the big drop of classification accuracy of subject b and g with small training sets.

**Table 9:** The mean classification accuracy and standard deviation (%) of 5 cross-validation (20% samples for training) with the number of corresponding channels (listed in the bracket) of COL and baselines on 5 subjects of DS1.

**Method**	**Subject**					**Mean**
	**aa**	**al**	**av**	**aw**	**ay**	
BS1	66.43	74.73	55.80	70.63	66.43	66.80
	± 3.90	± 1.21	± 2.42	± 2.63	± 2.31	
BS2	61.96	75.18	54.82	70.89	74.29	67.43
	± 3.75	± 7.10	± 5.45	± 3.32	± 3.48	
gsBLDA	64.64 (106)	80.18 (81)	56.70 (96)	73.75 (95)	68.84 (86)	68.82
	± 5.26	± 2.76	± 5.28	± 4.65	± 5.01	
MRCS	66.87 (115)	76.16 (71)	57.68 (49)	73.12 (75)	69.46 (33)	68.66
	± 5.61	± 1.08	± 2.15	± 3.21	± 1.94	
CSTI	64.73 (1)	89.64 (7)	59.82 (89)	68.30 (104)	85.80 (9)	73.66
	± 13.52	± 5.92	± 3.09	± 2.53	± 5.50	
NSGA-II	69.37 (47)	77.86 (39)	58.39 (45)	72.50 (60)	71.25 (34)	69.87
	± 4.20	± 2.29	± 2.31	± 3.44	± 1.47	
Proposed	**80.43** **(19)**	**90.88** **(14.8)**	**68.43** **(17.2)**	**94.13** **(15.6)**	**93.18** **(14.2)**	**84.81**
	± 3.34	± 3.81	± 5.29	± 2.15	± 2.00	

*The best performance is highlighted in bold. Performance of BS1 and BS2 indicates classification accuracy with all channels and 3C channels, respectively*.

**Table 10:** The mean classification accuracy and standard deviation (%) of 5 cross-validation (20% samples for training) with the number of corresponding channels (listed in the bracket) of COL and baselines on 4 subjects of DS2.

**Method**	**Subject**				**Mean**
	**a**	**b**	**f**	**g**	
BS1	63.38	55.37	59.00	62.00	59.94
	± 2.75	± 2.36	± 4.71	± 1.35	
BS2	57.00	52.75	57.50	63.00	57.56
	± 5.44	± 3.47	± 5.43	± 4.89	
gsBLDA	61.12(43)	57.37(36)	57.87(48)	63.88(54)	60.06
	± 3.96	± 6.00	± 2.88	± 1.68	
MRCS	63.38(59)	55.75(31)	60.25(24)	63.25(50)	60.66
	± 2.75	± 3.84	± 2.24	± 2.94	
CSTI	67.62(55)	58.25(48)	62.38(1)	71.62(58)	64.97
	± 3.99	± 5.56	± 3.01	± 3.82	
NSGA-II	64.87(24)	57.62(23)	60.25(20)	63.50(35)	61.56
	± 2.23	± 3.96	± 3.58	± 2.82	
Proposed	**81.75(14.2)**	**64.25(20.4)**	**78.88(15)**	**81.5(15.4)**	**76.59**
	± 4.04	± 11.49	± 8.79	± 6.50	

*The best performance is highlighted in bold. Performance of BS1 and BS2 indicates classification accuracy with all channels and 3C channels, respectively*.

### 4.3. Discussion

Simplified and well-generalized classification models are essential for the practical application of MI based BCI. Many efforts have been done in the feature selection and optimization of MI-EEG based on CSP, DSP, and SVM et al. However, due to the insufficient EEG data for model training in practical applications, the more training parameters a classifier requires, the more likely it tends to be overfitting, which reduces its practical value. In this study, we establish a L1 norm based channel optimization algorithm for MI-EEG classification. Compared with commonly used CSP methods, the parameters required for COL training are greatly reduced, contributing to a simplified and generalized classification model.

It is shown that the optimal channel distribution of the COL exhibit a physiologically interpretable topography, and the optimal frequency bands are mostly distributed around the μ and β rhythm, which is believed to be closely related to motor imagery. In addition, results on BCI competition datasets show that the COL maintains relatively high classification accuracy and F1 score with sparse features, indicating its good potential in practical applications. Further tests under small ratio of training samples show that the COL has good generalization performance, especially on DS1. As the training set ratio decreased from 80 to 20%, the average classification accuracy on DS1 changed from 85.86 to 84.81%, maintaining relatively high classification accuracy. The generalization of the COL algorithm benefits from the simplified model design and efficient extraction of motor-related features.

We further compared the classification performance under different reference conditions. The REST, an accurate zero-reference method, is applied to the multichannel EEG recordings. However, the classification performance is not significantly improved after REST processing compared to the simple CAR reference. We infer that the L1 norm-based sparse channel optimization and combination procedure may be insensitive to the detailed characteristics of the signal. As a result, the EEG signal obtained by the simple CAR preprocessing or the more accurate zero-reference preprocessing does not cause apparent difference in the classification performance.

There are still some limitations in this study. Firstly, the current study lacks theoretical and experimental proof of the convergence of COL. Secondly, this study only utilizes binary classification to evaluate the classification and generalization performance of the COL algorithm. In the future work, we will prove the convergence of COL theoretically and experimentally. More attention will be paid to flexible EEG processing and classification methods for improved channel-specific prediction accuracy. Further, novel embedding and interaction metrics for signals from multi-channels are also of great interest. And multi-classification problem will also be considered for improving the effectiveness of the COL algorithm in practical applications.

## 5. Conclusion

We introduced a novel method to optimize the features extracted from multichannel EEG by integrating inter-channel and intra-channel factors on motor imagery signal processing in this paper. Specifically, an *l*_1_-norm-based sparse regularized linear least square regression was introduced to learn a compact and accurate representation of MI-EEG. By maximizing the F-score of the EEG classification channel-specifically, COL discretely determines the optimal frequency bands for each channel independently. Simultaneously, by virtue of the sparse regularization term on channel weights, redundant and uninformative channels are discarded, while significant and task-relevant channels preserved. Subsequently, we designed an iterative algorithm to efficiently solve the constrained optimization problem and analyze its computational complexity. Experimental results on real world EEG datasets not only validated the effectiveness and efficiency of the proposed method compared with state-of-the-art methods but also provided convincing evidence of its feasible application in practical BCI systems.

## Author contributions

YZ and JH processed and analyzed the data, and wrote the manuscript; YC developed the l1-norm regularized method; HS, JC, and AK helped to acquire and interpret the data; YH and PZ helped data analysis; YZ helped in data interpretation and manuscript edit; JZ and CW supervised development of work, helped in manuscript edit and evaluation.

### Conflict of interest statement

The authors declare that the research was conducted in the absence of any commercial or financial relationships that could be construed as a potential conflict of interest.

## Footnotes

^1^In our proposed method, only **w** and *b* need to be trained. That means that only *C* + 1 parameters need to be trained, much fewer than existing approaches, including CSP. Thus, intuitively, the generalization of our method is better and it is probable to train our models with relatively small number of labeled samples. The experimental results in section4 demonstrate this hypothesis.

## Appendix

For any vector **x** ∈ ℝ^*n*×1^, the *l*_*p*_-norm is defined as

(A1) $$\begin{array}{l} ||\text{x}|{|}_{p}={\left(\overset{n}{\underset{i=1}{\sum }}|x_{i}{|}^{p}\right)}^{\frac{1}{p}}, \end{array}$$

where *x*_*i*_ is the *i*-th element of **x**. For any matrix **X** ∈ ℝ^*n*×*m*^, the *m*_*r*_ -norm is defined as

(A2) $$\begin{array}{l} ||\text{X}|{|}_{r}={\left(\overset{n}{\underset{i=1}{\sum }}\overset{m}{\underset{j=1}{\sum }}|{\text{X}}_{ij}{|}^{r}\right)}^{\frac{1}{r}}. \end{array}$$
